# Charcot knee — presentation, diagnosis, management — a scoping review

**DOI:** 10.1007/s10067-021-05775-8

**Published:** 2021-05-24

**Authors:** Victor Lu, James Zhang, Azeem Thahir, Andrew Zhou, Matija Krkovic

**Affiliations:** 1 School of Clinical Medicine, University of Cambridge, Cambridge, CB2 0SP UK; 2 Christ’s College, St. Andrew’s Street, Cambridge, CB2 3BU UK; 3 Department of Trauma and Orthopaedics, Addenbrooke’s Hospital, Cambridge, CB2 0QQ UK

**Keywords:** Arthrodesis, Charcot arthropathy, Knee, Total knee arthroplasty

## Abstract

**Background:**

Charcot arthropathy is a progressive, non-infectious, destructive inflammatory process. Charcot arthropathy of the knee (CK) is rare and diagnosis is often delayed, resulting in detrimental outcomes. This scoping review aims to investigate the literature on CK, present the pathognomonic features of CK to aid early diagnosis, and suggest gaps in the literature for future research.

**Methods:**

A systematic search of PubMed, EMBASE, Web of Science for literature relevant to CK was performed. Primary studies such as case reports, case series, retrospective studies were included. Review articles and animal studies were excluded.

**Results:**

Of the 513 results, 58 were included in qualitative synthesis. Average time from symptom onset to CK diagnosis was 50.5 months. Eighteen and twenty-one studies included patients who had diabetes mellitus and syphilis, respectively. Twenty-one studies reported pain as a presenting complaint, but the degree of pain didn’t correspond with the level of destruction. Oedema and joint effusion were noticed in 34 studies. Twenty-nine studies reported lower limb hypoesthesia and 17 studies reported decreased tendon reflex. Twenty-eight studies reported initial conservative treatment, often in a knee brace with minimal weight bearing, 9 of which needed subsequent surgical management. Twelve studies utilised arthrodesis, with fracture at the intramedullary nail entry site being the most common complication. Twenty-four studies utilised TKA.

**Conclusion:**

The literature on CK remains sparse, with most publications being case reports. Given that CK dramatically reduces quality of life, increases morbidity of patients, there is need for more literature on evidence-based options for early diagnoses and management.

**Supplementary Information:**

The online version contains supplementary material available at 10.1007/s10067-021-05775-8.

## Introduction

Charcot neuroarthropathy (CN) is the chronic, progressive, non-infectious destruction of bone and joints, in patients with peripheral neuropathy, as first described by William Musgrave in 1703 [1]. Charcot neuroarthropathy of the knee (CK) is a rare and under-researched area, resulting in considerable morbidity. However, it is a common foot and ankle related pathology, especially in those with diabetes mellitus.

Early diagnosis is important, and any patient with peripheral neuropathy, presenting with a red, warm, and erythematous knee joint, should be reviewed for CK. The cause of CK is wide-ranging, with patients often having many co-morbidities. In the past, syphilitic causes were common; however, as antibiotic therapy improved, diabetic causes are becoming more prevalent. Neurological causes, such as spinal canal stenosis and disk herniation, have also been reported.

Traditionally, conservative management with braces and limited weight bearing were common treatment options. In recent years, advancements in technology have allowed specific types of prosthesis to yield acceptable results. The paucity of literature and the dearth of large-scale clinical trials comparing the long-term outcomes following different treatment types could explain the lack of a robust management guideline for CK.

To our knowledge, there has not been any systematic or scoping review of the CK literature. This scoping review aims to systematically examine the literature regarding the presentation, diagnosis, management, and long-term outcomes, and point out gaps in the literature that could be filled with rigorously conducted CK studies that use a standard reporting template that we have proposed.

The manuscript does not contain clinical studies or patient data.

## Methodology

We conduced our review using the method described by Arksey and O’Malley [2].

### Identifying the research question

This scoping review aimed to answer the primary question: What is currently known about Charcot neuroarthropathy of the knee (CK)? Secondary questions were the following:

- What is the aetiology of CK?
- How does CK usually present?
- What are the pathognomonic features of CK?
- How is CK managed, and what complications could arise?

### Identifying relevant studies

In order to balance practicality with comprehensiveness and breadth, only English language studies, and literature formally published in sources such as journals were included. Before formulating our search criteria, an initial review of the literature was performed, to gauge the heterogeneity of this field, avoiding the chance of important studies being missed.

On March 2^nd^ 2021, a systematic search was performed on Embase, Medline, and Web of Science, which were considered comprehensive. The search strategy can be found in Online Resource 1.

### Study selection

After importing the studies into Mendeley reference manager, the in-built deduplication function was used. JZ and VL independently completed the title, abstract, and full-text screening, based on the inclusion and exclusion criteria. This was determined a priori based on the research questions. Agreement between authors was assessed for all studies and generated 78% agreement. A third reviewer (AT) was contacted for unresolvable disagreements.

Studies were included if they were conducted on human subjects (all ages, both sexes) and relevant to CK. Primary studies, such as case reports and retrospective observational studies were included, however those that only mention CK as a passing statement, with no further elaboration on specific outcomes measures were excluded. Review articles were also excluded, since they provide little first-hand quantitative or qualitative data for analysis, and different review articles could report on the same patients, thus duplicating data. For seven older studies, full text could not be found after an extensive search, and were excluded. The inclusion and exclusion criteria can be found in Online Resource 2.

### Charting and presenting the data

Data from each study were split into 4 different categories.

1. Demographics category includes number of patients in each study, ethnicity, mean age, gender, patient BMI, laterality of knee affected.
2. Presentation category included how the patient presented, time from symptom onset to CK diagnosis, any trigger for symptom onset, physical and neurological exam of the knee, joint aspiration, and histopathological outcomes.
3. Imaging category includes the pathognomonic features of CK seen on radiographs.
4. Treatment category includes a description of different treatment modalities, including complications encountered during follow-up, time to partial weight bear (PWB) and full weight bear (FWB), quantitative changes in knee range of motion and knee scores, and patient-reported outcome measures (PROMs).

Qualitative data were presented under the category to which they belong. Extracted quantitative data was analysed with IBM SPSS Statistics version 27. Statistical analyses focused on descriptive statistics such as mean, median and range.

The aforementioned review process was performed in accordance with Preferred Reporting Items for Systematic Reviews and Meta-analyses guidelines for scoping reviews (PRISMA-ScR) [3]. A PRISMA-ScR checklist is provided in Online Resource 3.

This scoping review was not prospectively registered in the International Prospective Register of Systematic Reviews PROSPERO.

## Results

### Search results

A total of 513 studies were identified in the original search, After de-duplication, 356 studies were identified for title and abstract screening. 239 studies were excluded, leaving 117 studies for full-text screening. Based on the inclusion and exclusion criteria, 58 articles were included for final analysis. Publication dates ranged from 1954 to 2020, the mean and median year of publication were 2003 and 2011, respectively. Online Resource 4 shows the PRISMA flow diagram.

The majority (n = 40) were single patient case reports, and 9 case series were included. The remaining 9 studies were retrospective studies investigating a group of CK patients treated at a single institute, the largest of which included 27 patients [4].

### Demographics

A total of 212 patients with 259 CKs were included, with the average age being 52.3 years, and 45.4% being male patients. 76.9% of all patients reported unilateral CK (33.8% right knee, 43.1% left knee). 23.1% of all patients reported bilateral involvement.

Patient BMI was only reported by 7 studies involving 71 patients, averaging 23.51 kg/m^2^ [4–10]. This is understandable given the aetiology of CK being different from other knee conditions such as osteoarthritis, for which weight is one of the greatest modifiable risk factors [11]. Patient ethnicity was reported by 8 studies involving 17 patients, mostly in those that were published in the twentieth century. Among them, 50% were Caucasian, 41.7% Afro-Caribbean origin, and 8.3% Asian [12–19]. Table 1 shows aetiologies of CK patients.

**Table 1:** Aetiology of patients with CK

Aetiology	Number of studies	Number of patients
Diabetes mellitus	**18** [4, 6, 8, 9, 16, 20–32]	**40**
Syphilis	**21** [5, 7, 9, 10, 17, 18, 28, 32–45]	**85**
Syringomyelia	**4** [9, 32, 46, 47]	**5**
Neurofibromatosis	**1** [48]	**1**
Familial amyloidotic polyneuropathy	**1** [19, 49]	**2**
Spinal cord injury	**6** [4, 50–54]	**9**
Riley-Dey syndrome	**1** [15]	**1**
Idiopathic sensory peripheral neuropathy	**1** [55]	**1**
Guillain–Barre´ Syndrome	**2** [4, 56]	**2**
Trauma-induced pseudogout	**1** [13]	**1**
Lacunar infarct	**1** [9]	**1**
Turner syndrome	**1** [4]	**1**
Charcot-Marie-Tooth disease	**1** [4]	**5**
Myelomeningocele	**1** [4]	**1**
Congenital insensitivity to pain	**1** [57]	**1**
Idiopathic	**7** [9, 12, 58–62]	**17**

### Presentation

All case reports mentioned how the patient presented initially, apart from two case series published in 1954 [14] and 1973 [37]. Retrospective studies were vaguer about the initial presentation of each patient. Among the 27 studies (68 patients) that reported time from symptom onset to diagnosis, on average, patients were diagnosed with CK 50.5 months after their symptoms occurred, which could be non-specific such as ambulatory disturbance, knee tenderness, gradual development of joint instability, with one patient diagnosed 20 years after the onset of walking difficulty [47]. Specific triggers are sometimes described, for example a misstep whilst hiking on the Appalachian Trail [60], or traumatic incidents [15, 23, 25, 35, 43].

- ***Pain***
  Twenty-one studies involving 52 patients described a degree of pain and tenderness as a presenting complaint [7, 16, 17, 19–24, 28, 35, 37, 38, 44, 47, 54, 57–61] especially during movement [21, 37, 57]. However, neuropathic arthropathy is classically described as a painless condition, with 9 studies involving 11 patients reporting an initial painless presentation [6, 8, 12, 13, 15, 36, 39, 41, 48]. Furthermore, the degree of pain does not correspond with the level of bone and joint destruction [19, 28, 37, 60, 62]. Some present with apparently unrelated symptoms, such as dysuria and sexual dysfunction, perhaps pathognomonic of syringomyelia which the patients also had [46, 47].
- ***Swelling***
  Oedema is commonly reported in CK patients and is often indicative of disease stage. As proposed by Eichenholtz, CK is divided into 3 phases, development, coalescence, reconstruction. 68 patients in 34 studies presented during the development or coalescence phase, and hence presented with swelling, joint effusion, erythema, and knee ballottement [5, 6, 8, 12, 13, 15–17, 19–23, 25–29, 35–37, 39, 40, 42–46, 48, 49, 52, 53, 59, 62]. One patient presented without oedema or erythema upon inspection, and radiographs confirmed that this patient’s CK was already beyond the fragmentation and coalescence stage at diagnosis [50].
  For patients presenting with these features, osteomyelitis, septic arthritis, pseudogout are important differentials, and 13 studies involving 16 patients performed joint aspiration studies to exclude them [12, 13, 16, 18, 23, 25, 27, 39, 42, 45, 49, 51, 58]. Among them, two reported a bloody aspirate [13, 45]. One case series reported 4 patients with staphylococcus pyogenes-mediated suppurative CK, diagnosed by a leukocyte count of > 100,000 cells/mm^3^ [18], and one reported presence of CPPD crystals in a patient with pseudogout-induced CK [13].
- ***Knee deformities***
  Valgus deformation was reported in 19 studies involving 114 patients [4, 7, 9, 12, 19, 28, 34, 35, 37, 38, 45, 49–51, 53–55, 57, 58] and varus deformation in 14 studies involving 34 patients [8, 16, 21, 24, 28, 30, 31, 34, 35, 37, 43, 53, 54, 62], often presenting as instability upon valgus and varus stress tests, or a positive Bohler test [52]. Ligamentous laxity and joint hypermobility were reported in 7 studies involving 14 patients [7, 19, 22, 26, 32, 50, 53]. This resulted in increased knee range of motion seen that decreased after treatment [34, 38, 44]. In two studies involving one patient each, the affected leg was shorter than the contralateral normal one [40, 48], producing a functional leg length discrepancy.
  Thickened, inflamed, indurated synovium was present in 11 studies involving 34 patients [5, 12, 20, 21, 23, 28, 35, 47, 52, 58, 60], with marked crepitus upon ambulation in 8 studies involving 30 patients [5, 7, 15, 21, 28, 35, 54, 62]. Five studies involving one patient each described knee joint or patella dislocation upon initial presentation [15, 42, 45, 54, 58]; these patients lost their proprioception and deep pain sensation, resulting in heightened stress experienced by knee joints. Together with synovial effusion stretching knee ligaments, this could result in joint dislocation [15, 42]. One patient in one case report presented with bone destruction so severe that it eroded the tibial tuberosity [29].
- ***Neurology***
  CK presents with characteristic neurological findings, with 29 studies involving 62 patients reporting lower limb hypoesthesia with diminished pain and temperature sensation [5–7, 13, 15, 17–20, 22, 27, 28, 31, 35–37, 39, 41–43, 45–47, 49–53, 55], and 6 studies with one patient each reported peripheral neuropathy [6, 7, 20, 50, 55, 58]. One patient presented with scars and burns due to lack of pain sensation [27]. Nevertheless, 5 studies involving 14 patients reported no sensory deficits [12, 15, 31, 35, 56]. Nerve conduction studies, performed with the monofilament test or EMG studies, produced pathological results such as chronic polyneuropathy with sensorineural loss [20, 50], and showed decreased nerve conduction velocity [40, 61]. Two studies involving one patient each performed biopsy of the sural nerve, and reported loss of myelinated fibres [40, 58]. Proprioception was diminished in 8 studies involving 30 patients [5, 7, 19, 28, 35, 41, 51, 52], perhaps leading to a positive Romberg sign seen in 4 of those studies involving 12 patients [35, 41, 51, 52]. Tendon reflex was reported as decreased or absent in the 17 studies involving 41 patients [7, 10, 15, 17, 18, 20, 28, 31, 35–37, 42, 43, 45, 49, 51, 52], but hyperactive in two studies involving 5 patients [37, 52].
- ***Histopathology***

Most studies did not perform histopathological analysis, yet those that did report findings pathognomonic for CK, such as hyperplastic inflamed synovium with bone and cartilage detritus [5, 9, 20, 28, 52] with fibrous pannus invasion, denoting a rapid breakdown of the joint [5, 21, 28]. Subchondral sclerosis with fibrous hyalinised tissue were reported by a five studies involving one patient each [7, 16, 20, 39, 52]. Histopathological analysis is key to pinpointing CK, differentiating it from isolated insufficiency fractures that could be related to osteonecrosis or chronic mineral imbalance.

### Imaging

Table 2 depicts the imaging findings on X-ray, CT and MRI modalities, and the prevalence of each imaging phenomenon.

**Table 2:** Number of studies presenting each imaging finding

	X Ray	MRI	CT
Tibial plateau destruction	**27** (49 patients) [8, 10, 13, 16, 20, 22–24, 26, 29–32, 35, 37, 39, 40, 43, 45–50, 52, 53, 62]	**8** (11 patients) [8, 16, 25, 27, 36, 46, 47, 52]	**11** (35 patients) [6, 19, 22–24, 26, 34, 35, 45, 46, 60]
Femoral condyle destruction	**10** (16 patients) [7, 15, 19, 27, 32, 39, 40, 48, 55, 58]	**2** (2 patients) [27, 36]	**2** (2 patients) [19, 46]
Knee joint effusion	**3** (6 patients) [17, 42, 43]	**7** (7 patients) [3, 12, 20, 27, 36, 46, 52]	**2** (2 patients) [20, 46]
Synovial thickening	**1** (1 patient) [17]	**3** (3 patients) [12, 20, 52]	**2** (2 patients) [20, 46]
Oedema	**1** (2 patients) [17]	**4** (6 patients) [8, 16, 23, 52]	**2** (2 patients) [6, 34]
Fragmentation	**9** (46 patients) [5, 7, 10, 12, 18, 29, 33, 37, 46]	**3** (5 patients) [9, 12, 25]	**1** (1 patient) [45]
Osseous Debris	**2** (4 patients) [5, 33]	**0**	**1** (1 patient) [46]
Joint space collapse	**7** (7 patients) [19, 27, 40, 51, 52, 57, 62]	**1** (29 patients) [9]	**0**
Calcium deposition in joint/Chondrocalcinosis	**6** (40 patients) [13, 15, 33, 35, 37, 53]	**1** (1 patient) [56]	**0**
Meniscal tear	**1** (1 patient) [48]	**1** (1 patient) [45]	**0**
Cruciate Ligament damage	**0**	**2** (2 patients) [45, 56]	**0**

Initial radiographic assessment was almost always conducted by X-ray, after which further scans in the form of CT or MRI are often performed, to get a more detailed visualisation of the joint, surrounding soft tissue, vasculature, and guide preoperative surgical planning.

The tibial-femoral (TF) angle is the angle between the anatomical axis of femur and tibia [63]. The normal knee alignment has a TF angle of 5–7° valgus, with a larger valgus angulation suggesting ‘knock-kneed’, and a smaller valgus angulation showing ‘bow-legged’. [64]. In six studies involving 47 patients, TF angle was reported as a radiological finding preoperatively [4, 34, 40, 49, 53, 55], and in two studies involving 26 patients, it was reported postoperatively [34, 38]. In general, the preoperative deformity caused varus deformity. The only study that compared pre- to post-operative angles reported an average correction of 11.7 varus to 6.1 valgus for 20 knees in 16 patients [34].

One case series involving two patients used single-photon emission computed tomography (SPECT) to confirm the diagnosis of CK, with increased uptake of tracer associated with the increased vascularity in the femoral condyles [26].

### Treatment and prognosis

Despite the low incidence of CK among lower limb pathologies, the high morbidity and rapid progression present difficulties in management and recovery. Current literature includes different treatment options; however, there is no universal treatment algorithm, perhaps due to lack of randomised control trails. The paucity of CK literature, and the difficulty in measuring treatment effectiveness makes it unlikely that any advocated treatment regimen will be standardised by the orthopaedic community in the near future. Furthermore, CK patients often have other co-morbidities such as syringomyelia, diabetes mellitus, which dictates a unique treatment pathway for each patient.

Length of follow-up was reported in 29 studies involving 135 patients [4, 5, 7, 9, 12, 14, 19–21, 23–26, 28, 30–32, 34, 38, 40, 44, 47, 51, 52, 54, 56–59]. Last follow-up was defined as the last interaction between patient and healthcare provider. The average was 4.5 years, with the longest follow-up being 14 years [59]. Time to PWB and FWB was reported in 10 studies involving 21 patients [10, 14, 23, 29, 30, 35, 43, 45, 54, 57] and 11 studies involving 20 patients [10, 14, 21, 23, 32, 43, 45, 46, 54, 57, 61], respectively, with the average time to PWB and FWB being 10.5 weeks and 15.1 weeks, respectively. Thirteen studies involving 95 patients reported knee scores [4, 5, 20, 28, 29, 32, 34, 38, 40, 51, 54, 55, 58], with the majority being American knee society score (AKSS) knee score and function score.

- ***Conservative treatment***
  Twenty-eight studies involving 68 patients chose conservative treatment (6,8,13,18,19,20,21,22,25,26,27,29,30,33,35,37,39,41,42,43,46,48,50,55,56,58,59,62). All used a combination of knee brace and consistent immobilisation. Conservative treatment was used for patients with a wide range of aetiologies, including 50% of studies with diabetic CK patients [6, 20–22, 25–27, 29, 30] whereby the possibility of functional improvement made procedures such as arthrodesis, which severely impacts post-operative mobility, unpopular. Custom made devices work best, considering each patient's unique pathological profile and lifestyle requirements [50]. Physical therapy [55] ameliorates patients’ quality of life, helping them adapt to a new lifestyle revolving around joint preservation.
  Pharmacological treatment is offered when there is a clear underlying cause. Underlying syphilis is often treated by antibiotics, with penicillin being most commonly used [18, 33, 34, 37, 41–43, 45]. Bisphosphonates were given in five studies involving six patients to reduce bone loss, slowing down the degenerative process [19, 25, 26, 50, 55]. Treatments aimed at reducing the pathological inflammatory process have also been used, such as NSAIDs [59], analgesia [19], indomethacin [13] and viscosupplementation [19].
  Among studies utilising conservative treatment, four involving 12 patients, and three involving one patient each reported time to PWB and FWB, respectively, with the average time being 19.1 weeks and 45.3 weeks, respectively. Four studies involving one patient each reported knee scores, with one case report using AKSS knee and function scores.
  Conservative management can be satisfactory to the patient [8, 22], with 7 patients in 7 studies being able to walk brace-free [13, 19–21, 25, 46, 50]. However, 9 case reports provide examples whereby this led to worsening of knee instability, progressive joint destruction, and severe gait disturbance, resulting in subsequent surgical management with total knee arthroplasty (TKA) [19–21, 25, 27, 29, 30, 42, 58].
- ***Arthrodesis***
  Twelve studies involving 27 patients [7, 12, 14, 17, 19, 21, 35, 40, 42, 45, 47, 57] opted for arthrodesis with aetiologies in 6 of them being syphilis [7, 17, 35, 40, 42, 45]. 7 of them [14, 17, 21, 35, 42, 47, 57] included patients treated in the twentieth century when prosthesis technology was limited, and less consideration was placed on long-term patient outcome measures such as functional mobility, thus the vastly limited range of motion post-arthrodesis was deemed acceptable. Majority of the literature regarding arthrodesis for CK management was published during the twentieth century. At best, patients were able to walk around pain-free with a quad cane [21], which allowed them to independently perform daily life activities [19], and even return to work [12]. Arthrodesis techniques include spring compression fixators [7], positive pressure fixators [17], intramedullary locking nail and bone grafting [19, 35]. Intramedullary nailing technique consists of the removal of the entire patella and synovium, followed by debridement of sclerotic tissue. With the newly formed joint surfaces at a 10–20° angle, the rod is inserted. The patient could only weight bear after radiographical fusion is confirmed [35].
  Among studies utilising arthrodesis, four involving 15 patients, and four involving 7 patients reported time to PWB and FWB, with the average time being 16.7 weeks and 21.7 weeks respectively. One case study used AKSS knee and function scores. Knee score improved from 25 to 85.
  The site of entry for intramedullary nails can be a fragile point prone to fracture [7, 14], causing fracture of the femoral shaft [38, 39] and fused knees with reduced mobility to become susceptible to traumatic injuries [17]. In general, arthrodesis complications were rare; in a case series of 9 patients [35], no metalwork failure was reported, however 3 patients developed an infection which resolved spontaneously over time.
- ***TKA***

Twenty-four studies involving 124 patients opted for TKA [4, 5, 9, 10, 20, 21, 25, 28–32, 34, 38, 40, 44, 51–54, 58, 60–62], with the majority published in the twenty-first century. Different levels of constraint are used to give the user differing levels of varus-valgus and flexion–extension stability, and is dependent on the degree of bone loss and ligamentous laxity. A range of prosthesis, such as highly constrained [21], semi constrained [51], rotating hinged [24], bilateral constrained [56], cemented [4] or uncemented [58] have shown to be effective in different circumstances. Autogenous bone grafts can be used to stabilise the prosthesis [31].

Among studies using TKA, four involving one patient each, and five involving 11 patients reported time to PWB and FWB, with the average time being 7.5 weeks and 10.4 weeks respectively. The Japan orthopaedic association score [51] and a hospital’s own score [58] were each used once. 11 studies involving 94 patients reported knee scores, with 4 studies involving 38 patients using AKSS knee and function scores [4, 5, 20, 54]. Average knee and function scores improved from 47.1 to 88.2 and 43 to 92.4, respectively. 10 studies involving 70 patients reported preoperative and postoperative range of motion, with an average improvement from 103.6 to 111.5. This degree of preoperative to postoperative improvement was less in magnitude compared to the knee score, with two studies reporting a slight decrease in range of motion post-operatively [34, 38].

Two patients in two case reports were treated initially with open reduction internal fixation, allowing fracture fixation away from the articular surface. However, both were complicated by delayed union and fixation failure, after which TKA was performed. There was one example of a failed knee osteotomy, although the disease course was complicated by a proximal tibia Schatzker type IV fracture from a previous traumatic incident [43]. One study reported a successful conservative treatment regimen consisting of immobilisation and knee-ankle–foot orthosis, after a failed TKA caused by prosthesis dislocation after progression of bone loss [8].

Due to the operation site, the peroneal nerve is at risk, and two case reports documented foot drop post-operatively due to nerve disturbance [20, 53]. Other complications include periprosthetic fracture [24], superficial [4, 9] and deep [5] infections, pressure ulcers [20], haematoma [9, 21], prosthesis loosening [4] and instability [9], leading to intra-articular heterotopic giant ossification in one patient, which resulted in an above-knee amputation [61]. In retrospective studies involving TKA as the primary management of CK, the complication rate is low. One study involving 16 patients reported no infection of dislocation, but one patient required a brace and cane to walk due to medial instability. No reoperation was needed, with a 100% substitution survival rate [34].

Only 8 studies involving 45 patients present PROMs, with 3 published in the twentieth century [21, 44, 58]. This includes whether the patient has returned to their pre-diagnosis level of mobility [9], what devices, such as frames and crutches, the patient requires [23], whether they are affected by any other Charcot joints, amount of pain patient is in [20], if daily activities are manageable [63], and how many ‘blocks’ the patient can walk without discomfort [63]. Especially with longer follow-up times and difficulty getting the patient back into clinic during COVID-19, patient reported outcomes such can be invaluable in gauging long-term impact on patients’ lives.

#### Discussion

CK is a rare condition that has been reported in the literature for many decades. With diverse aetiologies, the literature mostly consists of case reports, with a few small-scale retrospective cohort studies. To our knowledge, there has not been any randomised control trials, due to the ethical implications of not providing personalised treatment for such a complex and debilitating condition.

Diagnoses, particularly in the early stages, revolve around differentiating CN with other aetiologies that also produce erythematous swelling of the joint, such as osteomyelitis or an acute attack of gout. This is clearly important for management strategies, especially in those who go on to develop chronic CN with underlying osteomyelitis. Kucera et al. states that diagnosis should be ‘*based on a history of peripheral neuropathy*’ [30], which agrees with our findings, given that 62 patients in 29 studies reported lower limb hypoesthesia with diminished pain and temperature sensation.

Radiography is the preferred modality, although changes are typically delayed and have low sensitivity [65]. Especially in later stages, X-ray films show joint distention, dislocation, intra-articular debris, joint subluxation, and subchondral sclerosis [30], which agree with findings from Kucera et al. [30]. Such severe changes make it hard to differentiate bone marrow oedema of CN from underlying osteomyelitis [66]. Some authors propose the combined use of a Technetium scintigraphy and labelled leukocyte scans to diagnose an infected Charcot joint, with a 80–90% sensitivity and specificity [66, 67]. Nevertheless, radiography is still the most specific modality for CN diagnosis.

Eichenholtz published a radiological classification in 1966, comprising the following pathognomonic features of joint subluxation and bone debris [68], shown in Table 3. This was updated by Shibata et al., to reflect the fact that clinical signs of CN may precede radiological changes. Given the fact that initial CN findings involve inflammation, suggestions for an MRI-based classification system appeared. This would recognise the presence/absence of marrow oedema (active vs inactive), and presence/absence of cortical fractures (low vs high grade) [69]. This has the advantage of allowing early treatment, given the higher sensitivity of MRI over X-rays.

**Table 3:** Eichenholtz Classification—Temporal staging of CK based on the pathophysiological progression of the disease

Stage	Radiographic findings	Clinical findings
0 (prodromal)	Normal	Swelling, erythema, warmth
I (development)	Osteopenia, joint subluxation, dislocation	Swelling, erythema, warmth, ligamentous laxity
II (coalescence)	Absorption of debris, sclerosis, fusion of larger fragments	Decreased warmth, decreased swelling, decreased erythema
III (reconstruction)	Deformity consolidation, fibrous ankyloses, rounding and smoothing of bone fragments	Fixed deformity, absence of warmth, swelling, erythema

Conservative treatment used to be the main treatment for CN patients. First proposed by Henderson in 1905, those with joint deformity fared better with bed rest [70]. In 1971, Drennan et al. recommended complete rest to avoid joint breakdown [35]. Current literature supports non-weight-bearing; however, complete immobilisation brings upon its own risks, such as deep vein thrombosis, pressure ulcers, muscle atrophy. Conservative treatment using immobilisation with brace and minimal weight bearing has shown to be effective [8, 71], as it stops the vicious cycle that leads to destruction of the joint through repeated and unnoticed trauma. However, in most cases, this was the default option for patients who did not wish to undergo arthrodesis during the early stages of the condition [21, 29, 39], and is often insufficient to resolve the pathological destructive process [30].

Treatments aimed at decreasing the pathological inflammatory processes such as intra-articular corticosteroid injections have been used for Charcot’s arthropathy of the knees, shoulders, and hips. However, pain-free periods after the injection removes the protective barrier against self-injury, with excess activity and microtrauma leading to joint destruction [72]. Nevertheless, randomised controlled trails have been done to assess the outcome of bisphosphate therapy, and have found to reduce bone turnover and reduce the temperature of the affected joint [73].

Early surgical treatment usually involves arthrodesis; however, this severely reduces quality of life, especially for younger patients. Furthermore, varying cultures around the globe means that some groups such as the Japanese spend more time seated, making knee arthrodesis more debilitating [40]. Nevertheless, for elderly patients, arthrodesis can promote mobilisation and prevent extreme limb shortening [7, 19, 40].

Until relatively recently, TKA was contraindicated for knee involvement. The risk of incorrectly aligned prosthesis caused complication concerns, with the most common being periprosthetic fractures [24] and periprosthetic infections [4]. Cases of prosthetic dislocation were also common [8]. Nevertheless, with new implant technologies such as unconstrained, rotating hinged, and highly constrained prostheses [4, 32], recent studies show favourable outcomes after TKA management (5,25,32,40), and should be utilised especially for those with joint dislocation prior to TKA [30]. Yet, some mention an inferior outcome after TKA for knee joints treated in the earlier fragmentation stage, and recommend TKA only during the reconstructive or coalescence stages [9]. Furthermore, there are still contraindications for TKA, such as premature surgery [32], inexperienced surgeons [5], and rapidly deteriorating conditions [19]; however, with more research and experience, this method allows the best functional mobility and quality of life for patients.

### Limitations

The scoping review is a novel clinical research method for a rapid and easily-presentable way to map out the literature in a particular field, especially ones that are relatively underexplored and heterogenous, and uncover gaps in the literature where future systematic reviews could be conducted.

We used Arksey and O’Malley’s methodological framework for scoping reviews, which was relatively simple to follow. We covered three major multidisciplinary databases (Medline, Embase, Web of Science), which encompassed the vast majority of literature on Charcot knee. Indeed, we could not find any additional studies in the bibliography of included studies that were not found by our search. However, like systematic reviews, the search was performed on a certain day, mapping out the literature at a certain point in time, and very soon became out of date.

Some may regard scoping reviews as a ‘less rigorous’ systematic review, yet they have their unique differences. Systematic reviews aim to critically analyse a specific sub-section of the literature, whereas scoping reviews provide a broad overview, and categorise the literature by common subject matters, helping researchers efficiently identify where further data analyses can be carried out.

Scoping reviews are not meant to assess literature quality. The balance between breadth and depth is challenging, especially when the overall aim was to map out the current literature. It was not feasible to conduct a comprehensive assessment of literature quality given the large volume of studies identified. Furthermore, the studies included in this review spanned multiple decades, hence the reporting style for many outcome measures were heterogeneous, making it difficult for any rigorous systematic review to be performed.

As scoping reviews increase in popularity, it is important to have a recognised framework to ensure the high quality of reviews. The PRISMA-ScR checklist was thus utilised to ensure all aspects were covered, improving the transparency and quality of this scoping review.

## Conclusion

CK is rare, with current literature mostly limited to case reports. Given the significant morbidity involved, it’s paramount that prompt diagnosis is made, preferably with the more sensitive MRI scan during the ‘prodromal’ phase, to initiate early conservative treatment and maintain structural integrity. Leukocyte/marrow scintigraphy should be utilised to rule out any concomitant osteomyelitis.

Care must be taken with treatment, given the lack of consensus. Any management strategy should focus on joint and limb alignment, bone defect reconstruction, and ligament balancing. TKA should not be shunned, with recent literature showing its potential to increase quality of life. Given the wide range of causes of CN, it’s still unclear whether the underlying pathophysiology has an effect on long-term outcome.

A summary of the key findings is given in Table 4. Despite neuroarthropathy caused by diabetes mellitus affecting the feet more than the knee, the increasing prevalence of diabetes in the Western world will concomitantly increase the prevalence of CK, because diabetics are living longer, and neuropathic arthropathy is a late manifestation of autonomic neuropathy in diabetics [30, 74]. Due to the heterogeneity in reporting outcome measures between different studies, we propose a standard reporting template for future CK studies (Table 5).

**Table 4:** Summary of key findings

Topic	Key findings
Common aetiologies of Charcot Knee (in descending order)	Syphilis, Diabetes mellitus, idiopathic, spinal cord injury and syringomyelia
Biggest challenge to early diagnosis	Long delay from symptom onset to diagnosis due to non-specific nature of symptoms Traumatic injury usually acts as trigger for discovery after imaging
Common physical findings	Minimal pain, oedema of knee joint, valgus/varus deformity of knee, hypoesthesia, reduced/absent tendon reflex
Important differential diagnosis to rule out	Osteoarthritis and osteomyelitis
When is conservative treatment indicated	Diabetic causes when deformity was discovered early A clear underlying cause such as syphilis is present, whereby medication is indicated
When is arthrodesis indicated	Cases of failed TKA operations, such as periprosthetic infection cases When quality of life is significantly improved by pain reduction and increased mobility, and not diminished by the restriction in range of motion
When is TKA indicated	End-stage neuropathic arthropathy, especially the coalescence and reconstruction stages Those presenting with joint dislocation prior to surgery

**Table 5:** Proposed reporting template for future CK studies

Part	Item #	Checklist item
A-Demographics	1	Ethnicity
	2	Patient Age
	3	Patient Gender
	4	Patient BMI
	5	Laterality of knee affected
	6	Smoking status
B-Presentation	1	Aetiology
	2	Time from symptom onset to CK diagnosis
	3	Trigger for onset of symptoms (if any)
	4	Presenting complaint
	5	Physical exam of CK
	6	Neurological exam of affected lower limb
	7	Initial pre-treatment knee range of motion + knee scores
	8	Joint aspiration results (if appropriate)
	9	Any other Charcot joints present
C-Imaging	1	Primary diagnostic radiographical methodology (e.g. X-ray, CT, MRI)
	2	Radiographical findings
	3	Diagnostic scoring system using the Eichenholtz classification
D-Treatment	1	Treatment modality and reason describing the choice Detailed description of technique used, implants/devices used, and if a retrospective study, if it was performed by the same/different surgeon If technique is adapted/changed for a particular case, describe why and how
	2	Histopathological analysis of any intraoperative specimen taken
	3	Post-operative management of the patient, including when PWB and FWB is allowed^*^
	4	Time delays that may influence any time-dependent outcome needs to be described
	5	Post-operative knee range of motion + knee scores
	6	Any complications encountered during follow-up period, and if so, if revision surgery was needed
	7	Total follow-up time^*^
	8	PROMs including EQ-5D-5L, SF-36

^*^All time-dependent outcome measures must include a clear definition.

Currently, there is a lack of large-scale studies with adequate follow-up comparing treatment types or diagnostic imaging modalities between CK patients with similar baseline characteristics. There may be little appetite in surgeons for randomised control trials due to high complication rates and morbidity for patients, and low patient recruitment. PROMs provide a beneficial overview of lower limb functional recovery, and unfortunately were infrequently reported. Newer studies provide a more holistic picture of the patient’s quality of life post-treatment. Nevertheless, most only report anatomical and medical outcomes, rather than lifestyle factors. No study reported EQ-5D-5L or SF-36 indices. Normally, a scoping review could aid researchers to locate areas of the literature that could be further investigated with systematic reviews and meta-analyses. However we feel that this is premature, given the lack of high-quality large-scale studies in the literature conducted with similar baseline measures, and any systematic review would probably suffer from high heterogeneity.

## Supplementary Information

Below is the link to the electronic supplementary material.

Supplementary file1 (PDF 172 KB)

Supplementary file2 (PDF 168 KB)

Supplementary file3 (PDF 293 KB)

Supplementary file4 (PDF 184 KB)

## Data Availability

The authors confirm that the data supporting the findings of this study are available within the article [and/or] its supplementary materials.
