# Supplementary material for: Charcot knee — presentation, diagnosis, management — a scoping review
Source: Clin Rheumatol. 2021 May 24;40(11):4445–56. doi: 10.1007/s10067-021-05775-8 (PMC8143744; doi:10.1007/s10067-021-05775-8)
Supplement: Supplementary file 1 — Supplementary file1 (PDF 172 KB) [file 10067_2021_5775_MOESM1_ESM.pdf]

Charcot Knee - Presentation, Diagnosis, Management - A Scoping Review

Journal Name: **Clinical Rheumatology**

Victor Lu[†1], *James Zhang*[1], *Azeem Thahir*[2], *Andrew Zhou*[1], *Matija Krkovic*[2]

[1]*School of Clinical Medicine, University of Cambridge, CB2 0SP, United Kingdom*

[2]*Department of Trauma and Orthopaedics, Addenbrooke's Hospital, CB2 0QQ, United Kingdom*

[†] Corresponding author. Email: victorluwawa@yahoo.com.hk Postal address: *Christ's College, St. Andrew's Street, Cambridge, CB2 3BU*

***Online Resource 1*: Search strategy**					
**Embase via OVID**		**Medline via PubMed**		**Web of Science**	
Search term	Results	Search term	Results	Search term	Results
1. charcot.mp	10503	(("charcot"[All Fields] OR "charcot s"[All Fields] OR "charcots"[All Fields] OR "arthropathy, neurogenic"[MeSH Terms]) AND ("joint diseases"[MeSH Terms] OR ("joint"[All Fields] AND "diseases"[All Fields]) OR "joint diseases"[All Fields] OR "arthropathies"[All Fields] OR "arthropathy"[All Fields] OR ("osteoarthropathies"[All Fields] OR "osteoarthropathy"[All Fields]) OR "neuropath*"[All Fields]) AND ("knee"[MeSH Terms] OR "knee"[All Fields] OR "knee joint"[MeSH Terms] OR ("knee"[All Fields] AND "joint"[All Fields]) OR "knee joint"[All Fields])) NOT "Marie-Tooth"[All Fields]	**249**	(((charcot) AND ((neurogenic arthropathy) OR (arthropath*) OR (osteoarhtropath*) OR (neuropath*)) AND (knee) NOT (Marie-Tooth)))	**93**
2. neuropath*.mp	330837				
3. knee.mp	240548				
4. marie-tooth.mp	7062				
5. arthropath*.mp	33410				
6. osteoarthropath*.mp	4291				
7. "neurogenic arthropathy*".mp	56				
2 or 5 or 6 or 7	365845				
1 or 3 or 8	309				
9 not 4	**171**				
