# Supplementary material for: Charcot knee — presentation, diagnosis, management — a scoping review
Source: Clin Rheumatol. 2021 May 24;40(11):4445–56. doi: 10.1007/s10067-021-05775-8 (PMC8143744; doi:10.1007/s10067-021-05775-8)
Supplement: Supplementary file 2 — Supplementary file2 (PDF 168 KB) [file 10067_2021_5775_MOESM2_ESM.pdf]

Charcot Knee - Presentation, Diagnosis, Management - A Scoping Review

Journal Name: **Clinical Rheumatology**

Victor Lu[†1], James Zhang[1], Azeem Thahir[2], Andrew Zhou[1], Matija Krkovic[2]

[1]*School of Clinical Medicine, University of Cambridge, CB2 0SP, United Kingdom*

[2]*Department of Trauma and Orthopaedics, Addenbrooke's Hospital, CB2 0QQ, United Kingdom*

[†] Corresponding author. Email: victorluwawa@yahoo.com.hk Postal address: *Christ's College, St. Andrew's Street, Cambridge, CB2 3BU*

***Online Resource 2*: Inclusion and Exclusion Criteria**		
**Category**	**Inclusion Criteria**	**Exclusion Criteria**
Types of research	- Primary studies, including case reports, case series, retrospective observational studies.	- Studies based on secondary data obtained from primary studies or case reports.<br>- Review articles.
Types of publication	- Peer-reviewed research papers formally published in journals.<br>- Full conference abstracts published in journals.	- Letters to the editor<br>- Marketing material<br>- Dissertations/thesis.
Language	- English	- Non-English literature
Population	- Patients admitted to hospital for CK management.	- Animal studies<br>- Cadaveric studies
Dates	- All dates from the start of Medline, Embase, Web of Science	None
Outcome	- Any outcome measure pertinent to CK	- Studies that only mention CK as a passing statement, with no further elaboration on specific outcomes of those patients.
Others		- Studies whose full-text can't be found after extensive searches.
