# Supplementary material for: Charcot knee — presentation, diagnosis, management — a scoping review
Source: Clin Rheumatol. 2021 May 24;40(11):4445–56. doi: 10.1007/s10067-021-05775-8 (PMC8143744; doi:10.1007/s10067-021-05775-8)

Charcot Knee - Presentation, Diagnosis, Management - A Scoping Review

Journal Name: **Clinical Rheumatology**

Victor Lu[†1], *James Zhang*[1], *Azeem Thahir*[2], *Andrew Zhou*[1], *Matija Krkovic*[2]

[1]*School of Clinical Medicine, University of Cambridge, CB2 0SP, United Kingdom*

[2]*Department of Trauma and Orthopaedics, Addenbrooke's Hospital, CB2 0QQ, United Kingdom*

[†] Corresponding author. Email: *victorluwawa@yahoo.com.hk* Postal address: *Christ's College, St. Andrew's Street, Cambridge, CB2 3BU*

***Online Resource 4*: Preferred Reporting Items for Systematic Reviews and Meta-analyses (PRISMA) flow diagram of study inclusion**

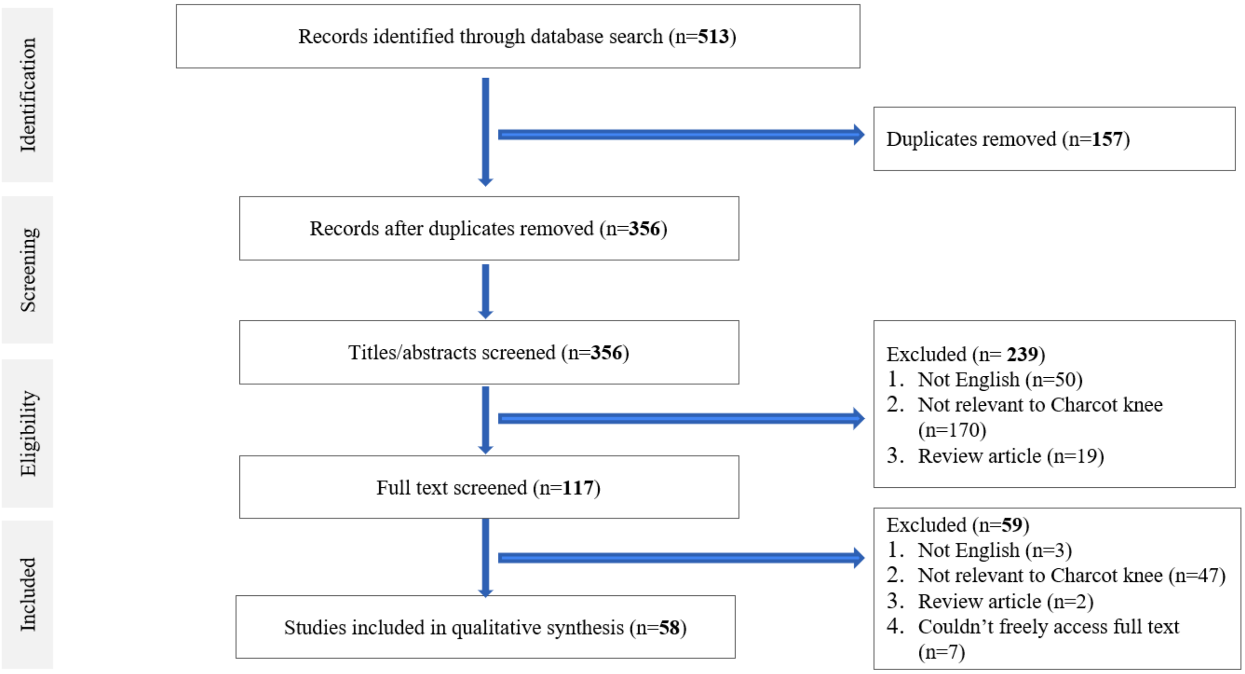

Supplement: Supplementary file 4 — Supplementary file4 (PDF 184 KB) [file 10067_2021_5775_MOESM4_ESM.pdf]
